# Association between Psychological Disorders, Mediterranean Diet, and Chronotype in a Group of Italian Adults

**DOI:** 10.3390/ijerph20010335

**Published:** 2022-12-26

**Authors:** Monica Dinu, Sofia Lotti, Antonia Napoletano, Abigail Corrao, Giuditta Pagliai, Marta Tristan Asensi, Vincenza Gianfredi, Daniele Nucci, Barbara Colombini, Francesco Sofi

**Affiliations:** 1 Department of Experimental and Clinical Medicine, University of Florence, 50134 Florence, Italy; 2 Department of Human Science, Georgetown University, Washington, DC 20057, USA; 3 Department of Biomedical Sciences for Health, University of Milan, 20133 Milan, Italy; 4 CAPHRI Care and Public Health Research Institute, Maastricht University, 6200 MD Maastricht, The Netherlands; 5 Nutritional Support Unit, Veneto Institute of Oncology IOV-IRCCS, Via Gattamelata, 64, 35128 Padua, Italy

**Keywords:** depression, anxiety, stress, Medi-Lite, Mediterranean diet, chronotype

## Abstract

Mental health conditions are a significant contributor to the global burden of disease. The aim of this study was to explore the association between psychological disorders, Mediterranean diet (MD), and chronotype. A total of 344 participants (74% women) with a mean age of 33.5 ± 13 years were recruited. According to the Depression Anxiety Stress Scale (DASS-21) score, 22% of participants had symptoms of depression, 23% anxiety, and 10% stress. The assessment of MD adherence through the Medi-Lite score revealed that participants with psychological disorders had significantly (*p* < 0.05) lower MD adherence than those without these conditions. Furthermore, less than 10% of the subjects with at least one symptom reported consuming the optimal amount of fruit and vegetables, while 72% showed excessive consumption of meat and meat products. Regarding chronotype, evening subjects reported the lowest MD adherence and the highest prevalence of all three psychological disorders analyzed. A multivariate analysis showed that female gender, age, being unmarried/single, sedentary lifestyle, and low MD adherence were associated with a significantly higher risk of depression, anxiety, and stress. Future studies are needed to explore the relationship between mental health and risk factors to improve personal and global population health.

## 1. Introduction

Mental health disorders are a significant contributor to the global burden of disease, at all sociodemographic levels [1,2]. In the past ten years alone, the estimated number of people suffering from depression has increased by 18.4% [3]. The substantial consequences of mental disorders, as leading causes of disability, pose challenges to both personal and population health [4], prompting a growing body of research surrounding potential risk factors. Among these factors, recent research has highlighted genetic, environmental, and lifestyle factors, such as diet, as determinants for mental health [5,6,7]. Studies have suggested that a higher adherence to the Western diet, and in particular a higher consumption of red meat, increases the risk of depression [7]. On the contrary, healthy diets, consisting of high levels of fruit, vegetables, and whole grains, may be associated with a decreased depression risk [8,9,10]. 

The Mediterranean diet (MD) is promoted as one of the healthiest dietary patterns for preventing non-communicable diseases (NCDs), including conditions involving cognitive impairment and mental illness [11,12]. These beneficial effects may be linked to the high consumption of plant-based products rich in polyphenols and vitamins, which are essential for brain function [13]. However, the MD is not isolated to a specific set of foods, and thus health benefits of the dietary pattern should be assessed in context with the lifestyle factors that accompany MD adherence. In this regard, there is increasing literature exploring the potential relationship between mental health and individual chronotype, a construct that reflects individual circadian rhythms and results in different patterns of behavior, including dietary patterns [14].

One of the most widely used methods to assess chronotype is the administration of validated questionnaires, such as the Morningness–Eveningness Questionnaire (MEQ) [15]. This tool allows for the classification of subjects into the morning chronotype, which is characterized by a tendency to fall asleep and wake up early, the evening chronotype, which is characterized by a tendency to be more active in the afternoon and evening hours, and the intermediate chronotype, which has intermediate characteristics [16]. Recent studies have suggested a relationship between the evening chronotype and affective disorders, anxiety, substance abuse, and depression risk [14,17,18]. Increasing evidence also suggests that individuals with the evening chronotype tend to have a poorer diet quality, lower adherence to the MD, and unhealthy dietary behaviors, unlike individuals with the morning chronotype [19,20]. Although there is a growing body of evidence exploring risk factors for mental health, including diet and chronotype, most research examines these factors separately. 

The aim of this study was to explore the association between psychological disorders, Mediterranean diet (MD), and chronotype in a group of Italian adults.

## 2. Materials and Methods

### 2.1. Study Design and Data Collection

Data were collected anonymously from May to July 2022 through an online questionnaire created with SurveyMonkey (www.surveymonkey.com, accessed on 31 August 2022) [21] and distributed among personal and non-personal contacts using social media networks. Prior to completing the questionnaire, eligible participants (≥18 years old) were asked to read the explanation of the study’s purpose. Participants were then asked to complete the first section of the questionnaire, containing sociodemographic and lifestyle information (gender, age, weight, height, attitude to smoking, marital status, education level, profession, frequency of physical activity, and medical history). Physical activity was evaluated with the question: “How often do you practice physical activity?” and the answers could be: “I do not practice it”, “I practice it 1-2 times a week”, or “I practice it 3 or more times a week”. Using the reported height and weight information, participants’ body mass index (BMI) was calculated (kg/m^2^). 

The study was conducted according to the principles of the Declaration of Helsinki, and it was approved by The Ethics Committee of the University of Florence, Florence, Italy (No. 199/2022, protocol 11917). Informed consent was obtained from all subjects involved in the study.

### 2.2. Assessment of Psychological Profile

Symptoms of depression, anxiety, and stress were determined using the 21-item Depression Anxiety Stress Scale (DASS-21) [22]. The DASS-21 is a self-reported questionnaire that includes 21 items, 7 items for each subscale: depression, anxiety, and stress. Responses to each statement are rated using a 4-point Likert scale, in which 0 corresponds to “I have never experienced” and 3 to “I have almost always experienced”. The depression subscale assesses symptoms associated with dysphoria and a loss of motivation and/or self-esteem. The anxiety subscale measures the symptoms of persistent anxiety and fear, including situational anxiety and autonomic arousal. The stress subscale investigates persistent excitement, irritability, and difficulty relaxing to assess non-chronic arousal. The DASS-21 has been validated in clinical and community populations compared to the standard Diagnostic and Statistical Manual of mental disorders (DSM-IV) structured clinical interview. It has also been shown to have excellent internal coherence in clinical populations (Cronbach’s α = 0.94 (DASS-Depression), 0.87 (DASS-Anxiety), and 0.91 (DASS-Stress)) [23]. In the present study, depression, anxiety, and stress were defined by the following scores: >9, >7, and >14, respectively [24].

### 2.3. Adherence to MD

The Medi-Lite adherence score, a 9-item questionnaire validated in 2017, was used to evaluate adherence to the MD [25]. The questionnaire evaluates consumption of nine food groups and categorizes consumption in accordance with the MD pattern. Daily consumption of fruits, vegetables, cereals, meat and meat products, dairy products, alcohol, and olive oil, and the weekly consumption of legumes and fish, are measured. 

Participants’ responses to the Medi-Lite questionnaire are scored using a point system based on three levels of consumption for each food group. A value of 2 is assigned to the highest category of consumption for foods typical of the MD (fruits, vegetables, cereals, legumes, and fish), while a 1 is assigned for intermediate consumption and 0 to the lowest category of consumption. Two points are assigned to regular olive oil consumption, one point for frequent consumption, and zero points for occasional consumption. For foods not typical of the MD (meat and meat products, dairy products), the value of 2 is assigned to the lowest category of consumption, 1 to the intermediate, and 0 to the highest category of consumption. A value of 2 is assigned to intermediate alcohol consumption (1–2 alcohol units/day), 1 to the intermediate category of alcohol consumption (1 alcoholic unit/day), and 0 points are assigned to the highest category of consumption (>2 alcoholic units/day). The final MD adherence score is obtained from the sum of the scores of the 9 categories and ranges from 0 (low adherence) to 18 (high adherence). 

### 2.4. Assessment of Chronotype

The final part of the questionnaire aimed to evaluate participants’ chronotype through the “Morningness–Eveningness Questionnaire” (MEQ) [15]. The MEQ consists of 19 multiple-choice questions that prompt the subject to think about normal patterns and behaviors throughout the day, such as: “At what time would you prefer to get up?”, “What time of day do you feel your best?”, and “What would be the best time to perform hard physical work?”. Scores range between 16 and 86 and categorize people into morning (59–86), intermediate (42–58), or evening (16–41) chronotypes. 

### 2.5. Statistical Analysis 

Data analysis was performed using the statistical package IBM SPSS Statistics for Macintosh, version 28.0 (IBM Corp., Armonk, NY, USA). Values of *p* < 0.05 were considered statistically significant. Continuous variables were presented as means ± standard deviations (SD), while categorical variables were presented as frequencies and percentages. Differences between two groups were estimated using the χ^2^ test for categorical variables and the Mann–Whitney test for continuous variables. The Kruskal–Wallis test was used to compare the differences between several groups for continuous variables. The Spearman correlation coefficient was calculated to assess the correlation between the DASS-21, the Medi-Lite score, and the MEQ.

To assess the association of different risk factors with symptoms of depression, anxiety, and stress, a logistic regression model was used. Results were reported as odds ratio (OR) and 95% confidence interval (CI). The considered risk factors for mental illness were gender, age, marital status, body weight, BMI, smoking habits, physical activity, MD adherence, and chronotype. All variables with a *p*-value < 0.05 in univariate analyses were included in the adjusted model (i.e., age and sedentary lifestyle for depression symptoms, sedentary lifestyle and lowest adherence to the MD for anxiety symptoms, and gender, age, and marital status for stress symptoms).

## 3. Results

### 3.1. Characteristics of Study Sample

A total of 344 participants with a mean age of 33.5 ± 13 years completed the questionnaire and were included in the analysis. The demographic, anthropometric, and lifestyle characteristics, according to gender, are shown in Table 1. Most participants were women (*n* = 254, 73.8%), unmarried (*n* = 205, 59.6%), and university graduates (*n* = 210, 61.1%). Based on the DASS-21, the prevalence of symptoms of depression was 22.1% (*n* = 76), anxiety 23.3% (*n* = 80), and stress 9.9% (*n* = 34). The mean Medi-Lite score was 10.2 ± 2.5, suggesting a moderate level of adherence to MD. Based on the MEQ score, 39% (*n* = 134) of the participants were classified as morning chronotype subjects, 53.5% (*n* = 184) as intermediate, and 7.6% (*n* = 26) as evening. Significant (*p*-value < 0.05) gender differences were observed in the prevalence of overweight/obesity, sedentary lifestyle, and stress symptoms. 

### 3.2. Psychological Disorders and MD Adherence

**Figure 1:**
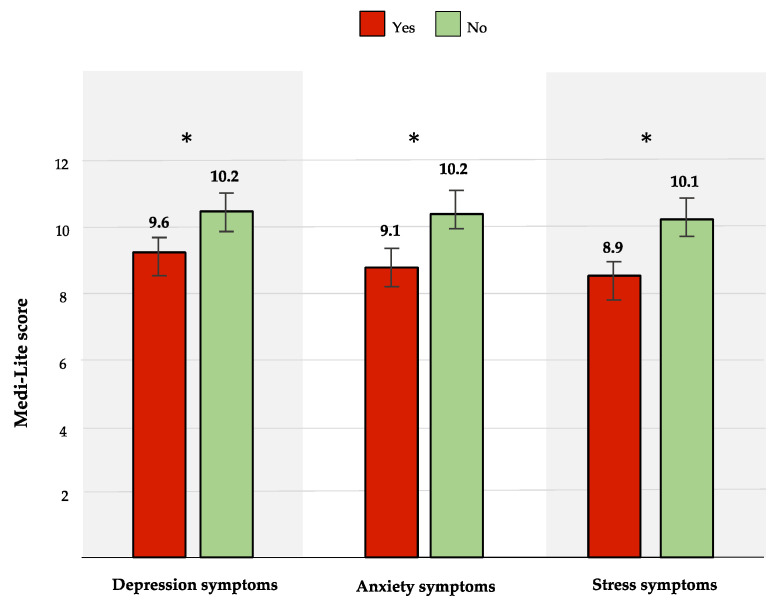
Medi-Lite values according to the presence (in red)/absence (in green) of symptoms of depression, anxiety, and stress, respectively (* *p*-value < 0.05).

A significantly lower adherence to the MD emerged in the three groups with depression, anxiety, or stress symptoms as compared to participants without these symptoms (Figure 1). 

In addition, small but significant negative correlations were found between the Medi-Lite score and the subscales of depression, anxiety, and stress (Figure S1).

**Figure 2:**
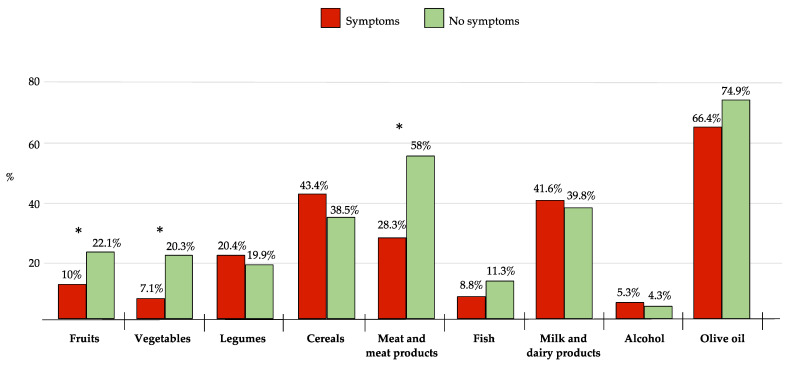
Percentage of participants reporting the optimal choice (i.e., the choice that gave 2 points) for the individual food components of the Medi-Lite score according to the presence (in red)/absence (in green) of symptoms of depression, anxiety, and stress (* *p*-value < 0.05).

The analysis of the optimal responses (i.e., the choice that gave 2 points) to the individual food groups composing the Medi-Lite score according to mental health status is shown in Figure 2. A comparison was made between subjects reporting no psychological disorders and subjects reporting at least one symptom among depression, anxiety, and stress. Subjects with at least one symptom reported significantly (*p*-value < 0.05) fewer optimal responses for fruit, vegetables, and meat and meat products. Specifically, less than 10% of participants with at least one symptom reported consuming more than 2 servings of fruit and more than 2.5 servings of vegetables per day, as recommended by healthy eating guidelines. In contrast, more than half (71.7%) of the participants that reported depression, anxiety, or stress symptoms showed excessive consumption of meat and meat products, with at least 1 serving per day.

### 3.3. Psychological Disorders and Chronotype

**Figure 3:**
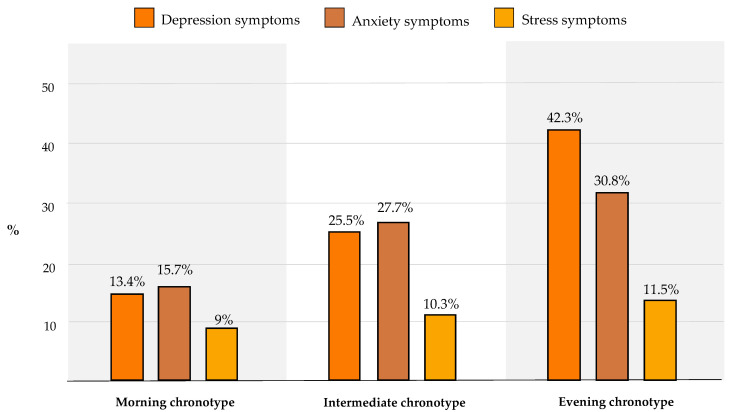
Percentage of participants reporting symptoms of depression, anxiety, and stress according to individual chronotype.

Figure 3 shows the percentage of participants reporting depression, anxiety, and stress symptoms according to individual chronotype. The prevalence of all three conditions increased linearly from the morning to the evening chronotype. The difference was particularly evident for depressive symptoms, which were reported by 13.4% (*n* = 18) of morning subjects and 42.3% (*n* = 11) of evening subjects. Similarly, only 15.7% (*n* = 21) of morning subjects reported suffering from anxiety symptoms, compared to 30.8% (*n* = 8) of evening subjects. 

Correlation analyses between the DASS-21 and the MEQ score revealed small but significant negative correlations for depression, anxiety, and stress subscales (Figure S2).

### 3.4. Chronotype and MD Adherence

Subjects with the evening chronotype not only had a higher prevalence of all psychological disorders, but also reported a significantly lower (*p*-value = 0.04) adherence to MD (9.6 ± 2.1) than the intermediate (9.7 ± 2.4) and morning (10.4 ± 2.3) chronotypes. Moreover, a positive, albeit very small, correlation was found between the MEQ and the Medi-Lite score (R = 0.15, *p*-value = 0.005). 

The analysis of the optimal responses to the individual food groups of the Medi-Lite score according to chronotype revealed significant differences (*p*-value = 0.01) only for the consumption of meat and meat products. Specifically, 69.3% (*n* = 18) of the evening subjects ate at least 1 portion of meat per day, compared to 57.6% (*n* = 106) of the intermediate and 40.3% (*n* = 54) of the morning subjects.

### 3.5. Regression Analysis

Finally, a regression analysis was performed to identify the risk factors associated with psychological disorders (Table 2). A significant association between being sedentary and the symptoms of depression (OR 2.31; 95% CI 1.32-4.06) and anxiety (OR 1.66; 95% CI 0.98-2.81) emerged from the univariate analysis. A significantly higher risk of anxiety symptoms was also observed in subjects less adherent to the MD, while being a woman and unmarried/single was significantly associated with a higher risk of stress symptoms. The results did not change after adjustment for possible confounding factors.

## 4. Discussion

To the best of our knowledge, this is the first study investigating the association between psychological disorders, adherence to the MD, and individual chronotype in a group of Italian adults. Participants with the analyzed symptoms of psychological disorders reported lower adherence to the MD, mainly determined by low consumption of plant-based foods and high meat consumption. Additionally, people with the evening chronotype showed the highest prevalence of depression, anxiety, and stress symptoms, and the lowest adherence to the MD. After performing a multivariate analysis adjusted for possible confounding factors, female gender, age, being unmarried/single, sedentary lifestyle, and low adherence to the MD were identified as risk factors for psychological disorders.

Mental illnesses account for one-third of disability-adjusted life years due to NCDs [4]. Depression and anxiety are the most prevalent mental disorders. While depression is characterized by anhedonia, depressed mood, and impaired cognitive function, anxiety is the feeling of fear experienced when faced with threatening or stressful situations. Anxious states are also closely associated with stress; under chronic stress conditions, the risk of severe anxiety increases as neurotransmitter synthesis is reduced [26]. In the present study, individuals with symptoms of depression, anxiety, and stress, as measured with the DASS-21, showed significantly lower adherence to the MD than subjects without these conditions. This is in line with the findings of many recent reviews on MD adherence and mental conditions, including the meta-analyses of Lassale et al. [9] and Nicolaou et al. [27], which showed a significant inverse association of the MD pattern with depressive symptoms. Similarly, a recent study of 50,000 Swedish women followed for 20 years found that the risk of depressive symptoms increased by 5% for every point less adherence to the MD at middle age [28]. These findings are supported when examined in a clinical context, as the results of recent randomized clinical trials demonstrate the beneficial role of the MD diet as a treatment for depression [29,30,31]. However, a mental disorder itself can be a major obstacle in following a healthy diet. Individuals exposed to greater stress conditions are more prone to unhealthy eating habits, which in turn may contribute to the development of symptoms of depression and anxiety [32].

In terms of individual food groups, our findings agree with those of Parletta and colleagues, who report low consumption of fruit, vegetables, legumes, whole grains, and high meat consumption amongst people with depression [30]. Interestingly, in the SMILES study—the first trial investigating whether improving diet quality improves symptoms of depression—participants assigned to follow a MD had a greater reduction in depressive symptoms, mainly due to the increased consumption of fruit, whole cereals, legumes, and olive oil [29]. The positive effects of the MD and plant-based foods on mental health are probably related to their protective functions against inflammation and oxidative stress [33]. Polyphenols inhibit proinflammatory cytokines, scavenge free radicals, and have neuroprotective action [13], while high-fiber foods have anti-inflammatory action through the maintenance of a healthy gut microbiota [34]. Vitamins and minerals also play a key role in supporting brain function. Folic acid deficiency in utero and childhood has been associated with an increased risk of depression in adulthood [35], while vitamin B6 and D deficiencies are implicated in the development of depression [26]. Findings from preclinical and clinical studies have also shown that deficiencies in vitamin C, magnesium, and zinc can cause anxiety, and supplementation can help alleviate anxious symptoms [36]. 

Although there is less extensive evidence examining the implications of the MD on anxiety and stress symptomology, our findings are in line with the inverse association between MD adherence and the risk for anxiety demonstrated by a cross-sectional study of Iranian adults [37]. A systematic review investigating the short-term effects of the MD on cognition and mental well-being also revealed that MD adherence results in improvement of mood, anxiety, and stress [38]. 

In this study, we also assessed chronotype, a construct that reflects individual circadian rhythms and results in different patterns of behavior, including dietary choices. Data revealed that evening subjects had significantly lower adherence to the MD than the other chronotypes, mainly due to high meat consumption. Furthermore, evening subjects also showed the highest prevalence of all measured psychological symptoms. These findings agree with previous studies suggesting a relationship between evening chronotype and increased anxiety and depression symptom severity [39,40,41,42]. The mechanisms governing the relationship between chronotype and the pathophysiology of depression, however, are still not fully understood. It has been hypothesized that the predisposition to depression observed in people with the evening chronotype may be due to a shift in the sleep–wake cycle. Indeed, evening individuals, who go to bed late at night and wake up late in the morning, are forced to adapt to social schedules that are not conducive to their natural biological rhythms. This discrepancy leads to sleep disturbances and an altered rhythmic activity of neurotransmitters, which may result in mental disorders [43]. The relationship between the evening chronotype and an increased risk of mental disorders could also potentially involve genetic mutations of specific clock genes [44]. 

An interesting finding of our study is the association observed in the multivariate analysis between female gender, age, being single/unmarried, sedentary lifestyle, and poor adherence to the MD and the risk of symptoms of mental disorders. Although there were no significant differences in the prevalence of depression and anxiety symptoms according to gender, being female was found to be significantly associated with an increased risk of stress. As suggested by Parker and Brotchie, gender could be a biological diathesis factor that predisposes women from puberty to be at greater risk of certain mental disorders [45]. A sedentary lifestyle may also be related to the onset of the symptoms of mental illnesses, but further investigation of temporality is important in evaluating this relationship [46]. In contrast, the regular practice of physical activity has shown effects comparable to the action of antidepressant and anxiolytic drugs [45], probably also due to the anti-inflammatory effects [47].

The present study has some limitations that need to be discussed. First, the assessment of psychological disorders was not based on a clinical diagnosis, but only on the severity of symptoms of mental disorders self-reported by the participants and calculated by the DASS-21 score. The use of questionnaires in this study that were based on self-completion may have led to misreporting and recall bias. Similarly, the technique used for chronotype determination may have been improved by using a more reliable biological test such as the dim-light melatonin onset (DLMO). While the questionnaire implemented in this study is validated and has benefits for ease of administration, the more expensive DLMO test is required to avoid inaccurate estimates of chronotype in future studies. Lastly, the cross-sectional nature of this study does not allow causality or temporality to be determined and the sample considered is not representative of the general population.

The current study also has some strengths as it provides new insights into the relationship between psychological disorders, dietary adherence, and chronotype, and identifies potential future lines of research. In fact, while previous research reveals lower MD adherence amongst evening subjects compared to other chronotypes, additional investigation is required to explore how the eating-related behaviors typical of the evening chronotype, including delayed meal consumption and the tendency to skip breakfast, mid-morning snack, and lunch, are involved in mental health outcomes of these subjects. The lifestyle factors responsible for meal timing and food choices, such as work/school schedules, should also be examined when assessing the relationship between dietary patterns and mental disorders. Finally, the order of the relationship between dietary patterns, chronotype behaviors, and risk factors that may predispose evening subjects to mental health conditions needs to be further examined.

## 5. Conclusions

In conclusion, our results showed that subjects with psychological disorders have lower adherence to the MD, mainly determined by low consumption of plant-based foods and high meat consumption. It was also observed that subjects with an evening chronotype showed the lowest level of MD adherence and the highest prevalence of depression, anxiety, and stress symptoms. Despite the limitations of the study, these results are interesting because they support current research on the hypothesized relationship between chronotype, MD adherence, and mental illnesses. Considering the burden of mental disorders and their substantial consequences, the relationship between chrono-nutrition and mental health must be further investigated to improve the personal and global health of the population.

## Figures and Tables

**Table 1:** Demographic, anthropometric, and lifestyle characteristics of participants in the total sample and according to gender.

	Total Sample (*n* = 344)	Men (*n* = 90)	Women (*n* = 254)	*p*-Value
**Age** (years) *	33.5 ± 13	31.5 ± 12.4	34.1 ± 12.9	0.05
**Marital status**				
Unmarried/single, n (%)	205 (59.6)	61 (67.8)	144 (56.7)	0.06
**Education level**				
University, n (%)	210 (61.1)	49 (54.4)	161 (63.4)	0.13
**Body weight** (kg) *	66.1 ± 14.4	78.8 ± 14.1	61.5 ± 11.4	<0.001
**BMI** (kg/m^2^) *	23.5 ± 4.3	25.1 ± 4	22.9 ± 4.2	<0.001
**Ponderal status**				
Overweight or obese, n (%)	89 (25.9)	36 (40.0)	53 (20.9)	<0.001
**Smoking habit**				
Yes	58 (16.9)	15 (16.7)	43 (16.9)	0.95
**Physical activity**				
Sedentary lifestyle, n (%)	112 (32.5)	17 (18.9)	95 (37.4)	0.001
**DASS-21 score**				
Depression symptoms, n (%)	76 (22.1)	16 (17.8)	60 (23.6)	0.25
Anxiety symptoms, n (%)	80 (23.3)	4 (4.4)	30 (11.8)	0.08
Stress symptoms, n (%)	34 (9.9)	15 (16.7)	65 (25.6)	0.04
**Medi-Lite score**	10.2 ± 2.5	10.0 ± 2.5	10.2 ± 2.5	0.40
**MEQ**				
Evening chronotype, n (%)	25 (7.6)	6 (6.6)	20 (7.8)	0.77
Intermediate chronotype, n (%)	184 (53.5)	51 (56.7)	133 (52.4)	
Morning chronotype, n (%)	134 (39.0)	33 (36.7)	101 (39.8)	

BMI = body mass index; DASS = Depression Anxiety Stress Scale; MEQ = Morningness–Eveningness Questionnaire. * Mean ± standard deviation

**Table 2:** Univariate and multivariate analyses of independent variables associated with psychological disorders.

	OR	95% CI	*p*-Value	aOR	95% CI	*p*-Value
Depression symptoms						
Age (years)	0.95	0.92-0.99	0.01	0.94	0.92-0.97	<0.001
Unmarried/single	2.90	1.56-5.38	0.22	-	-	-
Sedentary lifestyle	2.31	1.32-4.06	0.002	2.51	1.45-4-37	0.001
Lowest adherence to the MD *	1.35	0.79-2.32	0.25	-	-	-
Evening chronotype	2.06	0.87-4.88	0.15	-	-	-
Anxiety symptoms						
Age (years)	0.98	0.95-1.01	0.28	-	-	-
Smokers	1.64	0.84-3.17	0.14	-	-	-
Unmarried/single	1.57	0.76-3.23	0.21	-	-	-
Sedentary lifestyle	2.35	1.34-4.12	0.003	2.13	1.26-3.60	0.004
Lowest adherence to the MD *	1.66	0.98-2.81	0.05	1.69	1.01-2.84	0.04
Evening chronotype	0.94	0.36-2.44	0.91	-	-	-
Stress symptoms						
Woman	3.50	1.17-10.43	0.02	3.25	1.10-9.59	0.03
Age (years)°	0.94	0.90-0.99	0.01	0.94	0.90-0.98	0.01
Unmarried/single	3.22	1.34-7.70	0.009	3.13	1.31-7.46	0.01
Body weight (kg)	0.77	0.89-1.00	0.07	-	-	-
BMI (kg/m^2^)°	1.09	0.90-1.32	0.37	-	-	-
Lowest adherence to the MD *	1.79	0.86-3.72	0.11	-	-	-

BMI = body mass index; MD = Mediterranean diet. * Lowest tertile of MD adherence (total score < 9). Variables that differed significantly (*p*-value < 0.05) between subjects with and without symptoms of depression, anxiety, and stress were included in the univariate model. Age and BMI were used as continuous variables. Variables with a *p*-value < 0.05 in univariate analyses were included in the adjusted model (i.e., age and sedentary lifestyle for depression symptoms; sedentary lifestyle and lowest adherence to the MD for anxiety symptoms; gender, age, and marital status for stress symptoms).

## Data Availability

Additional data are available from the corresponding author upon reasonable request.

## Supplementary Materials

The following supporting information can be downloaded at: https://www.mdpi.com/article/10.3390/ijerph20010335/s1, Figure S1: Correlation analysis between Medi-Lite total score and depression, anxiety, and stress subscale scores; Figure S2: Correlation analysis between MEQ total score and depression, anxiety, and stress subscale scores.
