# Supplementary material for: Association between Psychological Disorders, Mediterranean Diet, and Chronotype in a Group of Italian Adults
Source: Int J Environ Res Public Health. 2022 Dec 26;20(1):335. doi: 10.3390/ijerph20010335 (PMC9819730; doi:10.3390/ijerph20010335)

Figure S2. Correlation analysis between MEQ total score and depression, anxiety and stress subscale scores.

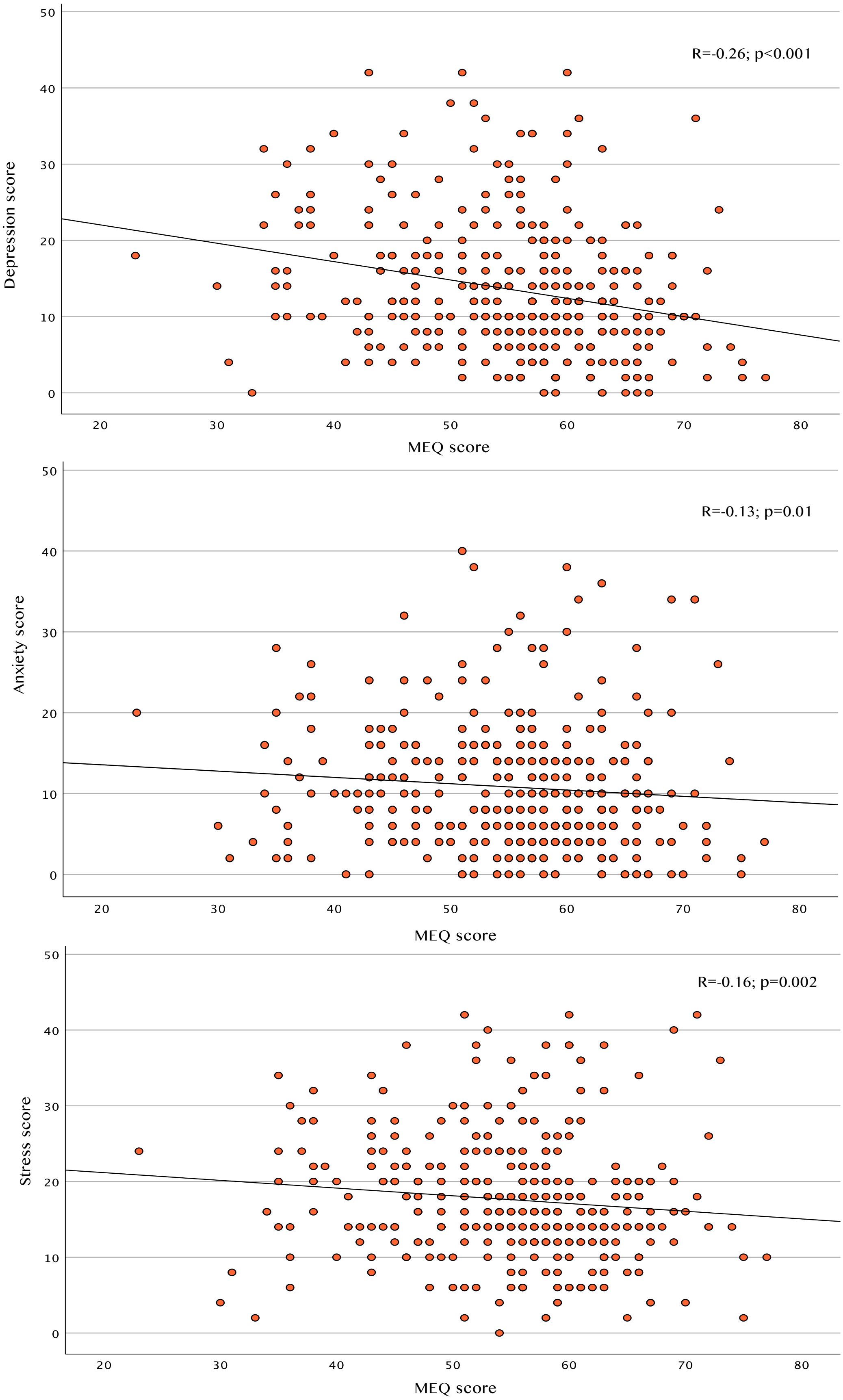

Supplement: Supplementary file 1 [file ijerph-20-00335-s001.zip › Supplementary figures/Figure S2.pdf]
